# Complete Genome Sequence of a Novel Genotype of Squash Mosaic Virus Infecting Squash in Spain

**DOI:** 10.1128/genomeA.01583-14

**Published:** 2015-02-12

**Authors:** Rugang Li, Shan Gao, Sven Berendsen, Zhangjun Fei, Kai-Shu Ling

**Affiliations:** a U.S. Department of Agriculture-Agricultural Research Service, U.S. Vegetable Laboratory, Charleston, South Carolina, USA; b Boyce Thompson Institute for Plant Research, Cornell University, Ithaca, New York, USA; c Rijk Zwaan Breeding B.V., De Lier, the Netherlands; d U.S. Department of Agriculture-Agricultural Research Service, Robert W. Holley Center for Agriculture and Health, Cornell University, Ithaca, New York, USA

## Abstract

The complete genome sequence of a new isolate of squash mosaic virus (SqMV) infecting squash plants in Spain was obtained using deep sequencing of small RNAs. The low nucleotide sequence identities, with only 87 to 88% for RNA1 and 84 to 86% for RNA2 to known SqMV isolates, suggested that this isolate belongs to a novel genotype.

## GENOME ANNOUNCEMENT

Squash mosaic virus (SqMV), of the genus *Comovirus* in the family *Secoviridae* (1), is a seed-borne virus infecting cucurbit crops worldwide (2–4). SqMV has a bipartite single-stranded RNA genome (RNA1 and RNA2) encapsidated separately with two capsid proteins. Both RNA molecules contain a genome-linked viral protein (VPg) and a poly(A) tail. Currently, two genotypes (or subgroups) that share only 88 to 89% genome nucleotide sequence identity have been recognized (2). In the present study, a new isolate (RZ-SqMV) collected in 2010 in Spain on a field for squash germplasm evaluation could not be detected by the real-time reverse transcription-PCR (RT-PCR) that was designed for SqMV (5), suggesting a greater genetic diversity than previously known among SqMV isolates. In order to obtain a full-genome sequence from the isolate RZ-SqMV, we employed deep sequencing of small RNAs (sRNAs) and assembly technology (6, 7). An sRNA library was constructed according to the published protocol (8) and sequenced in an Illumina HiSeq 2000. The sRNA sequence reads were processed and assembled based on the bioinformatics pipeline (7), resulting in two near-full genomes of RNA1 and RNA2. The authenticity of the newly assembled genomic sequences was confirmed through Sanger sequencing of additional RT-PCR products along the two genomic RNAs. The 5′ termini in both viral RNAs were obtained using rapid amplification of cDNA ends (RACE) technology. Upon treatment to a purified plant total RNA preparation with calf intestine alkaline phosphatase to dephosphorylate the RNAs and proteinase K to remove the VPg, 5′-adaptor ligation to the treated RNAs was performed either with a 5′-RACE adaptor from the Ambion FirstChoice RLM-RACE kit (Austin, TX, USA) or with the Illumina 5′-RNA adapter (5′-GUUCAGAGUUCUACAGUCCGACGAUC-3′). The 5′-terminal sequences obtained by RACE for RNA1 and RNA2 were in agreement using either ligation adaptor. The two SqMV RNAs began with an identical consensus sequence (UAUUAAA), in agreement with the 5′-terminal sequences in other comoviruses, except the Chinese isolate of SqMV, which is reported to have a unique terminal end (4). The complete genome sequence for the isolate RZ-SqMV was obtained. With a total of 5,858 nucleotides (nt), excluding a poly(A) tail, RNA1 contains a single open reading frame (ORF) encoding a polyprotein of 1,858 amino acids (aa). RNA2 has 3,370 nt encoding two overlapping polyproteins (1,009 aa and 920 aa, respectively) initiating either at nucleotide position 219 (AUG219) or 486 (AUG486) and terminating at position 3248 with a UAG stop codon. Using the complete genome sequences of RNA1 and RNA2, RZ-SqMV was found to share only 87 to 88% sequence identities in RNA1 and 84 to 86% sequence identities in RNA2 to all known SqMV isolates from both recognized SqMV genotypes. Such low levels of sequence identity suggest that RZ-SqMV should be considered a third genotype of SqMV. The complete genome sequence obtained from this novel genotype will allow us to better understand the genetic diversity in SqMV and to improve the molecular-based detection.

### Nucleotide sequence accession numbers. 

The full genomic sequences of RZ-SqMV were deposited in GenBank under the accession numbers KP223323 and KP223324.
